# Primary Angiosarcoma of the Breast: A Single-Center Retrospective Study in Korea

**DOI:** 10.3390/curroncol29050267

**Published:** 2022-05-04

**Authors:** Yeon-Jin Kim, Jai-Min Ryu, Se-Kyung Lee, Byung-Joo Chae, Seok-Won Kim, Seok-Jin Nam, Jong-Han Yu, Jeong-Eon Lee

**Affiliations:** Division of Breast Surgery, Department of Surgery, Samsung Medical Center, Sungkyunkwan University School of Medicine, Seoul 06351, Korea; yeonjin.kim@samsung.com (Y.-J.K.); jaimin.ryu@samsung.com (J.-M.R.); sekyung.lee@samsung.com (S.-K.L.); bj.chae@samsung.com (B.-J.C.); seokwon1.kim@samsung.com (S.-W.K.); seokjin.nam@samsung.com (S.-J.N.)

**Keywords:** primary angiosarcoma of the breast, breast angiosarcoma, primary sarcoma, angiosarcoma

## Abstract

Due to the rarity of primary angiosarcoma of the breast, optimal management is based on expert opinion. The aim of this study was to review all primary angiosarcomas of the breast obtained from a single center in terms of clinicopathologic characteristics, treatment, and survival outcomes. From 1997 to 2020, 15 patients with primary angiosarcoma of the breast underwent either mastectomy or wide excision. We analyzed the clinicopathologic data to assess disease-free survival and overall survival. Fifteen women with primary angiosarcoma of the breast were identified. The mean age at diagnosis was 33 years (range: 14–63 years). The overall mean tumor size was 7.7 cm (range 3.5–20 cm). Upon histological grading, there were three cases of low grade, five intermediate grade, six high grade, and one unidentified grade. The five-year disease-free survival rate was 24.4%, and the five-year survival rate was 37.2%. The survival rate of the low-grade patient group was statistically higher than that of the intermediate- or high-grade patient groups (*p* = 0.024). Primary angiosarcoma of the breast is a rare aggressive tumor characterized by high grade and poor outcome. Histologic grade appears to be a reliable predictor of survival. There are no standard treatment guidelines; thus, optimal R0 surgical resection remains the best approach. The roles of neoadjuvant, adjuvant chemotherapy, and radiotherapy remain unclear.

## 1. Introduction

Angiosarcoma of the breast is a rare entity with poor prognosis, comprising less than 1% of all soft-tissue tumors [1,2,3]. Breast angiosarcoma commonly is divided into two types, primary and secondary angiosarcoma. Primary angiosarcoma of the breast develops de novo with no prior breast radiation. It occurs within the breast parenchyma, usually affecting women in their 30s to 50s [2,4]. Secondary angiosarcoma of the breast occurs in the setting of radiation therapy as part of breast-conservative treatment of breast cancer and is typically seen in older patients. [1,4].

Primary angiosarcoma of the breast is rarer than secondary angiosarcoma and has no known risk factors [5]. It usually is derived from the endothelial cell lining of the vascular channels and does not involve the regional lymph nodes [6]. However, angiosarcoma is aggressive and tends to have a high risk of local and distant metastases [1,7].

Due to the rarity of these tumors, optimal management is based on expert opinion. Complete surgical resection with optical margins (R0 resection) is the most common treatment [2]. The best surgical methods for resection are uncertain due to lack of long-term outcome data comparing wide excision and mastectomy.

The role of radiotherapy and chemotherapy remains unclear. Some studies have insisted that radiotherapy before surgery is not recommended, and that adjuvant radiotherapy conveys better local control [8,9]. However, one study showed no effect of radiotherapy on overall survival [10]. According to the meta-analysis study, it was revealed that adjuvant radiation therapy after surgery for primary angiosarcoma of the breast had a statistically significant effect on recurrence-free survival [2]. A prior study showed that adding chemotherapy to the treatment of angiosarcoma has a significant benefit on reduced risk of local recurrence [11]. However, other studies showed that adjuvant chemotherapy has no statistically significant benefit for breast angiosarcoma [2,12]. The effectiveness of the adjuvant treatment is uncertain.

The aim of this study was to review all cases of primary angiosarcoma of the breast diagnosed from 1997 to 2020, in a single center, and to describe a single-institution experience with primary angiosarcoma of the breast, including clinicopathologic characteristics, treatment, and survival outcomes.

## 2. Materials and Methods

This retrospective study included 15 patients with primary angiosarcoma of the breast who were treated at Samsung Medical Center from 1997 to 2020, accessed through the electronic medical recoding system of the institute. This study was approved by the institutional review board (Approval number: 2021-09-037) of the Samsung Medical Center.

We reviewed the demographic data, tumor size, histologic grades, treatment modality, and survival data. Tumor size was defined as the largest dimension recorded on the pathology report. If excisional biopsy was performed and followed by operation at Samsung Medical Center, the largest length was recorded by adding to the previous excision size. Tumor grade was categorized as low, intermediate, or high.

Overall survival (OS) was measured from the date of surgery to the date of last follow-up or the date of death, as recorded in Statistics Korea records. Disease-free survival was measured from the date of surgery to the date of any recurrence or death. Overall survival and disease-free survival (DFS) were evaluated using the Kaplan–Meier method with the log-rank test. All statistical analyses were carried out using IBM SPSS v 27.0 (SPSS, Inc., Chicago, IL, USA).

## 3. Results

From 1997 to 2020, 15 patients who were diagnosed with primary angiosarcoma of the breast were treated at Samsung Medical Center. All patients presented with a palpable mass and were diagnosed with a core needle biopsy. Radiologic imaging such as via mammograms, ultrasound and MRI, was performed for patients

All cases were defined as primary angiosarcoma without prior diagnosis of breast cancer or radiation treatment. All patients were female, and the mean age at diagnosis was 33 years (range: 14–63 years).

The overall mean tumor size was 7.7 cm (range 3.0–25 cm). For histological grade, there were three patients of low grade, five of intermediate grade, six of high grade, and one unidentified grade (Table 1).

Thirteen patients underwent mastectomy, eight of whom also received axillary surgery (Table 2). However, no node metastasis was present in the axillary surgery group. Wide excision was performed in only two patients (13.3%). Surgical margin was negative in all patients.

Recurrence was detected in 10 patients (66.7%). We described the site of the first recurrence. Some patients were found to have distant metastasis after first local recurrence. The median follow-up period was 29 months (5.6–89 months). The last follow-up was observed in July 2021. Local recurrence occurred in four patients and local contralateral breast recurrence was observed in two patients. Distant recurrence was noted in three patients and one who had both local and distant recurrence. In distant metastasis, one was pulmonary, three were bone metastases (Table 2 and Table 3). Wide excision was performed in patients with local recurrence and palliative chemotherapy was performed in patients with distant metastases. Two patients with contralateral breast recurrence underwent wide excision and one patient with synchronous local and distant metastases received palliative chemotherapy (Table 2).

As shown in Table 2, one patient was diagnosed with angiosarcoma on both sides and underwent bilateral breast surgery.

In terms of adjuvant therapy after surgery, three patients received both chemotherapy and radiation therapy, five patients received radiation therapy only, and one patient received chemotherapy alone. Only one patient underwent mastectomy after neoadjuvant chemotherapy (Table 2). The adjuvant chemotherapy regimen in Samsung Medical Center was Adriamycin combined with alkylating agents (ifosfamide), followed by taxane agent (paclitaxel) or Adriamycin combined with alkylating agents (cyclophosphamide). The pediatric chemotherapy regimen in the center was etoposide combined with ifosfamide.

**Figure 1:**
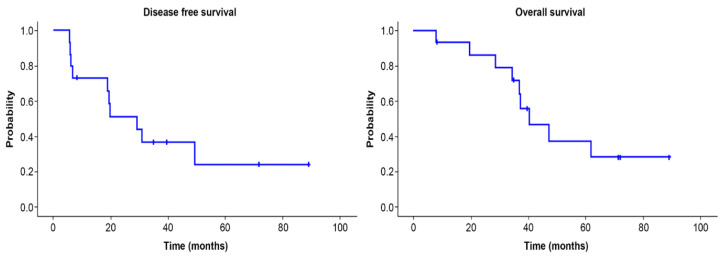
Disease-free survival and overall survival of primary angiosarcoma of the breast. The 5-year disease-free survival rate was 24.4% and the 5-year survival rate was 37.2%.

**Figure 2:**
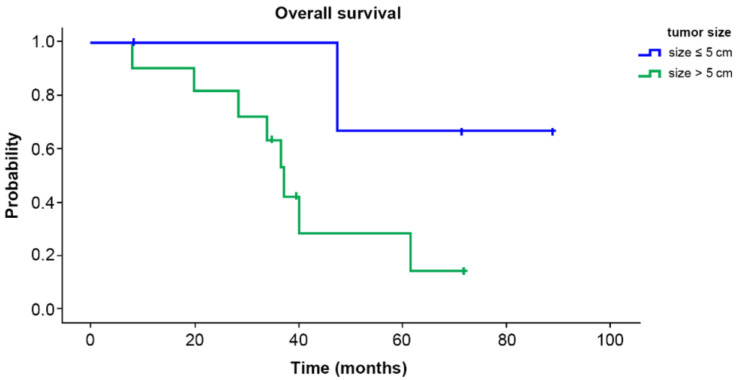
Overall survival according to tumor size. The 5-year survival rate was 28.3% in the group with tumor size ≥5 cm and 66.7% in the group with tumor size <5 cm. There was no significant difference between groups (*p* = 0.096).

Overall survival and disease-free survival are shown in Figure 1. The five-year survival rate was 37.2%, and the five-year disease-free survival rate 24.4%. Overall survival according to tumor size is shown in Figure 2. The five-year survival rate was 28.3% in the group with tumor 5 cm or more in size and 66.7% in the group with tumors smaller than 5 cm. There was no significant difference (*p =* 0.096).

**Figure 3:**
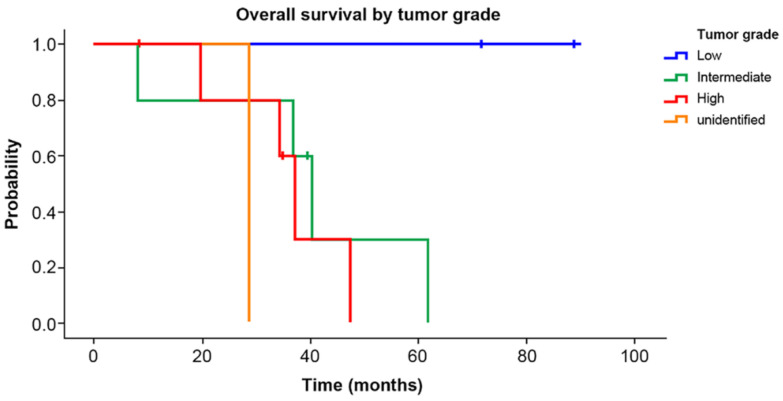
Overall survival by tumor grade. The 5-year survival rate was 100% in the low-grade group, 30% in the intermediate-grade group, and 0% in the high-grade group. The survival rate of the low-grade group was significantly higher than that of the intermediate- and high-grade groups (*p* = 0.024).

Overall survival by tumor grade is shown in Figure 3. The five-year survival rate was 100% in the low-grade group, 30% in the intermediate-grade group, and 0% in the high-grade group. The survival rate of the low-grade patient group was statistically higher than that of the intermediate- or high-grade patient groups (*p* = 0.024)

**Figure 4:**
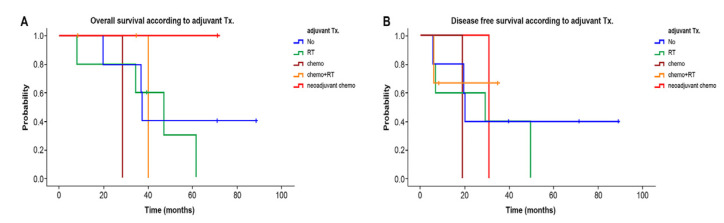
Overall survival (**A**) and disease-free survival (**B**) according to adjuvant treatment. There were no significant differences in 5-year overall survival (*p* > 0.05, Figure 4A) and 5-year disease-free survival (*p* > 0.05, Figure 4B) between groups according to adjuvant treatment. Abbreviations: adjuvant Tx, adjuvant chemotherapy; RT, radiotherapy; chemo, chemotherapy; neoadjuvant chemo, neoadjuvant chemotherapy.

Figure 4A,B shows the overall survival and disease-free survival according to type of adjuvant 15 patients, including one patient with neoadjuvant chemotherapy.

At the time of last follow-up, six patients were alive without distant metastatic disease. Only one of the 6 patients experienced local recurrence and was alive until the last follow-up (Table 3).

## 4. Discussion

As in previous studies [2,4,5,13], primary angiosarcoma of the breast occurs in younger females between 30 and 50 years and can arise de novo with no risk factors. Primary angiosarcoma of the breast usually develops in the lining of the endothelial cell of the vascular channels and often involves the breast parenchyma without triggering factors [6]. Therefore, angiosarcoma appears mostly as a palpable mass, and the age at diagnosis is lower than the average age for invasive breast cancer [5]. This is consistent with our study. We found the average age at diagnosis of primary angiosarcoma of the breast was 33 years, which is younger (range: 14–63 years) than that of invasive breast cancer occurring in the 40–49-year age group, according to the Korea Breast Cancer Society registry data (KBCS) [14]. The minimum age of onset of primary angiosarcoma of the breast was 14 years in our study.

Several studies reported breast angiosarcoma as a more aggressive malignancy of the vascular endothelium, and the overall prognosis is poor compared to that of other invasive breast cancers [15,16]. In our study, the five-year overall survival rate of primary angiosarcoma of the breast was 37.2%, while the five-year overall survival rate of invasive breast cancer was 93.2% according to the KBCS [14].

Several studies suggested that the grade seemed to be the most consistent prognostic factors for primary angiosarcoma of the breast in regard to both OS and DFS [2,17,18]. In total, 6 of the 15 patients had high-grade disease on histopathology, and the median overall survival was 40 months (range: 8.2–71.6 months). We revealed a significantly higher survival rate of low-grade tumor than that of intermediate or high grade (*p* = 0.024). Other studies reported that histological grade was associated strongly with clinical presentation and overall prognosis. They noted an improved DFS for low- and intermediate-grade tumors compared to high-grade ones [18,19,20].

Several studies have suggested that tumor size is a prognostic factor [2,15,18,21,22]. Other studies have also revealed increased risk of local recurrence and decreased overall survival with larger tumor size [2,18,22]. In contrast to those studies, our study showed lower survival rates in groups with larger tumor sizes, though the difference was not significant (28.3% for size ≥ 5 cm vs. 66.7% for < 5 cm, *p* = 0.096). Although we did not find statistical difference of survival rate related to tumor size due to our small sample size, we did find the trend of the difference in survival rate according to size.

In terms of adjuvant treatment, survival was associated not favorably with administration of adjuvant chemotherapy or radiation therapy in our study. Other studies reported unclear roles of neoadjuvant and combined adjuvant chemotherapy and radiotherapy [13,19]. However, one author suggested that adjuvant radiotherapy can reduce local recurrence [1]. Another author reported that tumor size > 5 cm can predict patients at higher risk of local recurrence, who are more likely to obtain benefit from adjuvant radiation therapy [23]. In the analysis from one study, adjuvant radiation therapy seemed to have a significantly positive impact on recurrence-free survival when both primary angiosarcoma of the breast and secondary angiosarcoma of the breast were analyzed together. Despite concerns about radiation-induced etiology and complications in the re-irradiation environment, this study found that the local recurrence rate in primary and secondary angiosarcoma was lower when patients received surgery and adjuvant radiotherapy; this was in contrast to the lack of significant difference reported in our study [2]. So, the role of adjuvant radiotherapy remains controversial. Additionally, it was reported that chemotherapy is beneficial in high-grade lesions and in the metastatic setting [15]. Based on the results of previous studies, the lack of an association of survival with adjuvant therapy in the present study might be due to the retrospective study design and the relatively small number of patients.

The best treatment for primary angiosarcoma of the breast is surgery with R0 resection [2,18,22]. In our study, 13 patients underwent mastectomy, and only two underwent wide excision, both of whom had negative resection margins. One study revealed that patients who underwent breast-conserving surgery did not have worse prognosis than those who underwent mastectomy [24].

The role of axillary lymph node dissection in primary angiosarcoma of the breast is unknown, as breast angiosarcoma is due primarily to hematogenous spread [25]. According to one study, all 13 patients who underwent axillary staging showed absence of involved nodes. There also was no node metastases in the patients with axillary staging in our study. Based on these results, axillary surgery is not suitable in patients with breast angiosarcoma.

This study is limited by the very small sample of breast angiosarcoma and retrospective nature of this analysis, which prevents any definite conclusions. Some findings that failed to reach statistical significance might be due to lack of statistical power.

## 5. Conclusions

In conclusion, breast angiosarcoma is a rare aggressive tumor characterized by high grade and poor outcome. Histologic grade appears to be a reliable predictor of survival. There are no standard treatment guidelines, and optimal R0 surgical resection remains the best approach. The roles of neoadjuvant, adjuvant chemotherapy, and radiotherapy remain unclear.

## Figures and Tables

**Table 1:** Patient Demographics and Characteristics (*n* = 15).

Clinicopathological Features		No. of Patients (%)
Age		
Median (range)	33 years (range 14–63 years)	
Grade	Low	3 (20.0)
	Intermediate	5 (33.3)
	High	6 (40.0)
	Unknown	1 (6.7)
Tumor size (cm)	>5 cm	11 (73.3)
	≤5 cm	4 (26.7)
Operation	Mastectomy	13 (86.7)
	Wide excision	2 (13.3)
Adjuvant chemotherapy	Yes	4 (26.7)
	No	11 (73.3)
Adjuvant Radiotherapy	Yes	8 (53.3)
	No	7 (46.7)

**Table 2:** Summary of Cases (*n* = 15).

Patient	Age	Grade	Tumor Size (cm)	Surgery (Date, Type)	Adjuvant Chemotherapy	Adjuvant Radiotherapy	Recurrence	Treatment of 1st Recurrence
1	35	3	6.0	27 November 1997 Lt. Total mastectomy	No	Yes	Local (Lt. chest skin)	Wide excision
2	31	2	10.0	27 October 1999 Rt. Total mastectomy	No	Yes	Distant (Bone)	Palliative chemoTx.
3	29	3	4.2	02 December 1999 Lt. Total mastectomy	No	Yes	Local (Lt. chest skin)	Palliative chemoTx.
4	19	2	11.2	27 February 2001 Rt. Total mastectomy	No	Yes	Local (Rt. chest skin)	Wide excision
5	21	2	10.0	02 April 2004 Lt. Total mastectomy +ALND	No	No	Distant (Bone)	Palliative chemoTx.
6	44	1	3.5	24 December 2009 Lt. wide excision	No	No	No	
7	28	2	8.0	30 March 2010 Rt. Total mastectomy +ALND	Yes AI # 4 + paclitaxel # 4	Yes	Local contralateral breast (Lt. chest skin)	Wide excision
8	14	unidentified	25.0	17 February 2011 Rt. Total mastectomy	Yes EI (# 45)	No	Distant (Bone)	Palliative chemoTx.
9	47	3	5.5	22 April 2011 Rt. Total mastectomy +ALND	No	No	Local contralateral breast (Lt. chest skin)	Wide excision
10	63	1	1.0	20 August 2013 Rt. wide excision	No	No	No	
11	14	3	9.0	28 February 2014 Lt. Total mastectomy + SLNBx, Rt. Wide excision	No	No	Local +Distant (Lt.chest skin, Lung)	Palliative chemoTx.
12	47	1	5.5	25 August 2015 Lt. Total mastectomy +ALND	Neoadjuvant Tx. AC # 4+ D # 4	No	Local (Lt. chest skin)	Wide excision
13	43	2	5.5	21 December 2017 Rt. Total mastectomy + SLNBx	No	Yes	No	
14	25	3	7.5	24 August 2018 Rt. Skin sparing mastectomy +SLNBx	Yes AC # 4	Yes	No	
15	41	3	3.0	22 May 2020 Lt. Total mastectomy + SLNBx	Yes AC # 4	Yes	No	

Abbreviations: ALND, axillary lymph node dissection; SLNBx, sentinel lymph node biopsy; Tx, treatment; A, Adriamycin; C, Cyclophosphamide; D, Docetaxel; I, ifosfamide; E, Etoposide.

**Table 3:** Outcomes of Primary Breast Angiosarcoma (*n* = 15).

Outcomes		No. of Patients (%)
Recurrence	Local	4 (26.7)
	Local contralateral breast	2 (13.3)
	Distant	3 (20.0)
	Local + Distant	1 (6.7)
	No recurrence	5 (33.3)
Survival	Alive	6 (40.0)
	Death	9 (60.0)

## Data Availability

The data presented in this study are available on request from the corresponding author.
